# Hematopoietic stem cell transplantation in children with chronic granulomatous disease: the Spanish experience

**DOI:** 10.3389/fimmu.2024.1307932

**Published:** 2024-02-01

**Authors:** Laura Alonso García, David Bueno Sánchez, Jose Maria Fernández Navarro, Alexandra Regueiro Garcia, Miguel Blanquer Blanquer, Maria Isabel Benitez Carabante, Yasmina Mozo del Castillo, Jose Luis Fuster Soler, Maria Luz Uria Oficialdegui, Luisa Sisinni, Antonio Perez Martinez, Cristina Diaz de Heredia Rubio

**Affiliations:** ^1^ Servicio de Hematología y Oncología Pediátricas, Hospital Universitari Vall d´Hebron, Barcelona, Spain; ^2^ Servicio de Hemato-Oncología Pediátrica, Hospital Universitario La Paz, Madrid, Spain; ^3^ Sección de Oncología y Hematología Infantil Hospital Universitari i Politècnic La Fe, Valencia, Spain; ^4^ Departamento de Hematología y Oncología Pediátricas Hospital Clínico Universitario de Santiago, Santiago de Compostela, Spain; ^5^ Unidad de Trasplante Hematopoyético y Terapia Celular, Hospital Clínico Universitario Virgen de la Arrixaca, Instituto Murciano de Investigación Biosanitaria (IMIB), Murcia, Spain

**Keywords:** chronic granulomatous disease, children, hematopoietic stem cell transplantation (HCT), graft failure, graft versus host disease, immunodeficiency

## Abstract

**Introduction:**

Hematopoietic stem cell transplantation (HCT) can cure chronic granulomatous disease (CGD). However, transplant-associated morbidity or mortality may occur, and it is still controversial which patients benefit from this procedure. The aim of this retrospective study was to evaluate the outcome of pediatric patients who received HCT in one of the Spanish pediatric transplant units.

**Results:**

Thirty children with a median age of 6.9 years (range 0.6–12.7) were evaluated: 8 patients received a transplant from a sibling donor (MSD), 21 received a transplant from an unrelated donor (UD), and 1 received a haploidentical transplant. The majority of the patients received reduced-intensity conditioning regimens based on either busulfan plus fludarabine or treosulfan. Relevant post-HCT complications were as follows: i) graft failure (GF), with a global incidence of 28.26% (CI: 15.15–48.88), 11.1% in patients with MSD (1.64–56.70) and 37.08% in unrelated donors (19.33–63.17); and ii) chronic graft-versus-host disease (GVHD), with an incidence of 20.5% (8.9–43.2), 11.1% in patients with MSD (1.64–56.70) and 26.7% in unrelated donors (10.42–58.44). Post-HCT infections were usually manageable, but two episodes of pulmonary aspergillosis were diagnosed in the context of graft rejection. The 2-year OS was 77.3% (55.92–89.23). There were no statistically significant differences among donor types.

**Discussion:**

HCT in patients with CGD is a complex procedure with significant morbidity and mortality, especially in patients who receive grafts from unrelated donors. These factors need to be considered in the decision-making process and when discussing conditioning and GVHD prophylaxis.

## 1 Introduction

Chronic granulomatous disease (CGD) is a rare primary immunodeficiency, included in group V of the UIUS classification (1), and is associated with phagocyte dysfunction resulting from genetic defects in some of the different subunits of the NADPH oxidase complex. Depending on the protein subunit affected, the pattern of inheritance is autosomal recessive (AR) or X-linked. As a consequence of the molecular defect in the respiratory burst, patients develop not only recurrent infections, typically bacterial or fungal, but also immune dysregulation and inflammation, particularly colitis and chronic lung disease. Improved diagnosis, understanding of the physiopathology, lifelong antimicrobial prophylaxis, immunosuppression, and prompt treatment of complications have increased the survival of these patients over the last few decades, and CGD patients are now expected to survive into adulthood. However, despite antimicrobial prophylaxis, infections still occur and the lifetime risk of invasive *Aspergillosis*, the leading cause of death, is estimated to be 20%–40%. In addition to a compromised survival, patients with CGD who remain on conventional treatment also suffer a significant disease burden. They experience failure to thrive and have an impaired quality of life. A higher median survival rate has been reported for autosomal recessive forms compared with X-linked forms (50 versus 38 years) (2, 3).

Hematopoietic cell transplantation (HCT) is currently the only potentially curative therapy available for CGD. However, it is controversial whether all CGD patients can benefit from HCT, as the procedure is associated with potentially life-threatening complications. In more recent years, transplant-related morbidity and mortality have been reduced by improvements in low-toxicity conditioning protocols, supportive therapy, management of infections, and graft-versus-host disease (GVHD), leading to increased interest in early HCT for CGD patients. In addition, a reduction in the number of episodes of infection and a decrease in the rate of growth retardation have been reported after a successful HCT (4, 5). In these circumstances, the indications for transplantation in CGD have become less restrictive. Unrelated and mismatched donors can be considered acceptable, and patients with few complications are considered candidates. This situation has recently led to an increase in the number of CGD patients in our HCT units.

This study aimed to evaluate the situation in Spain regarding the number of pediatric CGD patients receiving HCT and their outcomes.

## 2 Patients and methods

The study is a retrospective review of pediatric patients who underwent an HCT between 2007 and 2020 in five centers of the Spanish Pediatric Group for Hematopoietic Stem Cell Transplantation and Cellular Therapy.

Patients or their legal guardians had previously signed an informed consent for stem cell transplantation and for the data registry. Approval was obtained from the Ethics Committee for Clinical Research.

Patients admitted to HCT transplant units received supportive care and antimicrobial prophylaxis according to institutional guidelines. All patients received antifungal prophylaxis, either lyposomal amphotericin B-based or azole-based. Prophylactic acyclovir was given to patients who were seropositive for HSV, VZV, or CMV. CMV reactivation was routinely monitored, and treatment was given if reactivation occurred. Antibacterial prophylaxis was not given routinely.

For conditioning therapy, recommendations from EBMT, ESID, and IEWP guidelines were routinely followed where available (6).

### 2.1 Definitions

Neutrophil recovery was defined as the first of 3 consecutive days with an absolute neutrophil count >0.5 × 10E9/L; platelet recovery was defined as unsupported platelet count >20 × 10E9/L.

Primary graft failure was defined as failure to achieve neutrophil recovery or donor chimerism <5% on day +28 post-HCT.

Secondary rejection was defined as graft loss after neutrophil engraftment, as evidenced by decreasing donor chimerism with or without accompanying pancytopenia.

Grading of acute GVHD (aGVHD) was performed according to the Glucksberg-Seattle criteria and the NIH consensus criteria for chronic GVHD (cGVHD) (7–10).

In the situation where busulfan AUC was not available, the intensity of the conditioning regimen was determined according to whether the dose had been prescribed according to protocol A (myeloablative, busulfan-based) or protocol C (reduced intensity, busulfan-based) of the ESID/IEWP guidelines (6).

### 2.2 Statistical methods

Quantitative variables are expressed as means and standard deviations and qualitative variables as absolute numbers and percentages. For continuous variables, statistical tests are performed using Student’s *t*-test or Mann–Whitney’s U test, depending on the distribution and the variable. Chi-square or Fisher’s exact test is used for qualitative variables. The general characteristics of the patients grouped by transplant are described, and statistical tests are performed that show homogeneous populations with a level of significance greater than 0.05. Kaplan–Meier estimation was used to calculate the survival and cumulative incidence of the various endpoints. The log-rank test was used to test for differences among groups for these time-to-event variables.

## 3 Results

Data from 30 pediatric patients with CGD who underwent HCT between 2007 and 2020 at five transplant centers were analyzed. The characteristics of the patient, the transplant, and the outcomes are detailed in **Supplementary Table 1** and summarized in **Table 1**.

**Table 1:** Summary of patient and transplant characteristics.

	Number of patients
Sex	
Male	23
Female	7
Inheritance	
X-linked	14
AR	12
Missing data	4
Donor	
MSD	8
10/10 UD	13
9/10 UD	8
Haploidentical	1
Conditioning	
Busulfan, fludarabine	21
Busulfan, cyclophosphamide	1
Treosulfan, fludarabine	3
Treosulfan, thiotepa, fludarabine	5
Serotherapy	
ATG	11
Alemtuzumab	15
No	4
*In-vitro* T-cell depletion	
No	25
TCRαβ/CD19 depletion	2
CD45 RA+ depletion	1
Partial CD3 depletion	2
Stem cell source	
Bone marrow	23
Peripheral blood	7

AR, autosomal recessive; MSD, matched sibling donor; UD, unrelated donor; ATG, antithymocyte globulin.

Twenty-four male and six female patients were included. One patient had associated McLeod syndrome. The female patients had an AR inheritance pattern. The median age at diagnosis was 1.2 years (IQR 1 month–9 years) and the median age at transplantation was 6.9 years (IQR 0.6–12.7 years). Nine patients were ≤4 years old at HCT. All patients had experienced bacterial or fungal infections prior to HCT, and 18 of them also had inflammatory episodes. However, while all patients had significant complications prior to HCT, these were not active at the time of HCT in the majority of patients, with only two patients reporting active inflammation. Relevant pulmonary sequelae of previous complications were described in two of these patients.

Residual oxidative burst capacity was reported in seven patients; in the remaining patients, this complication was either missing, reported as “absent,” or 0%.

One patient received a transplant from a haploidentical donor, eight patients from matched sibling donors (MSD), and 21 patients from unrelated donors. The unrelated donors were 10/10 HLA matched in 13 cases and 9/10 HLA matched in eight cases.

The most common conditioning regimen was based on busulfan (*n* = 22): 21 patients received busulfan and fludarabine, and one patient transplanted in 2009 received busulfan and cyclophosphamide. The AUC of busulfan was assessed in nine patients. The AUC value was 85–95 mg*h/L in one patient and 65 mg*h/L in the remaining patients. The eight patients received treosulfan-based conditioning, either treosulfan plus fludarabine (*n* = 3) or treosulfan, thiotepa, and fludarabine (*n* = 5). Seven patients received peripheral blood stem cell (PBSC) transplants, and the rest of them received bone marrow (BM). *Ex-vivo* graft T-cell lymphodepletion (TCD) was performed in five patients, four of whom received grafts from 9/10 HLA-matched donors. The TCD consisted of CD19/TCRαβ^+^ cell depletion in two cases, partial depletions of the total CD3 cell dose in two patients, and CD45RA^+^ cell depletion in one patient. For the patient who received a haploidentical transplant, the post-transplant cyclophosphamide platform was selected.

All patients except one received grafts with a cell content above 2 × 10^8^ CN/kg or 2 × 10^6^ CD34/kg.

### 3.1 Outcome

#### 3.1.1 Engraftment

**Figure 1:**
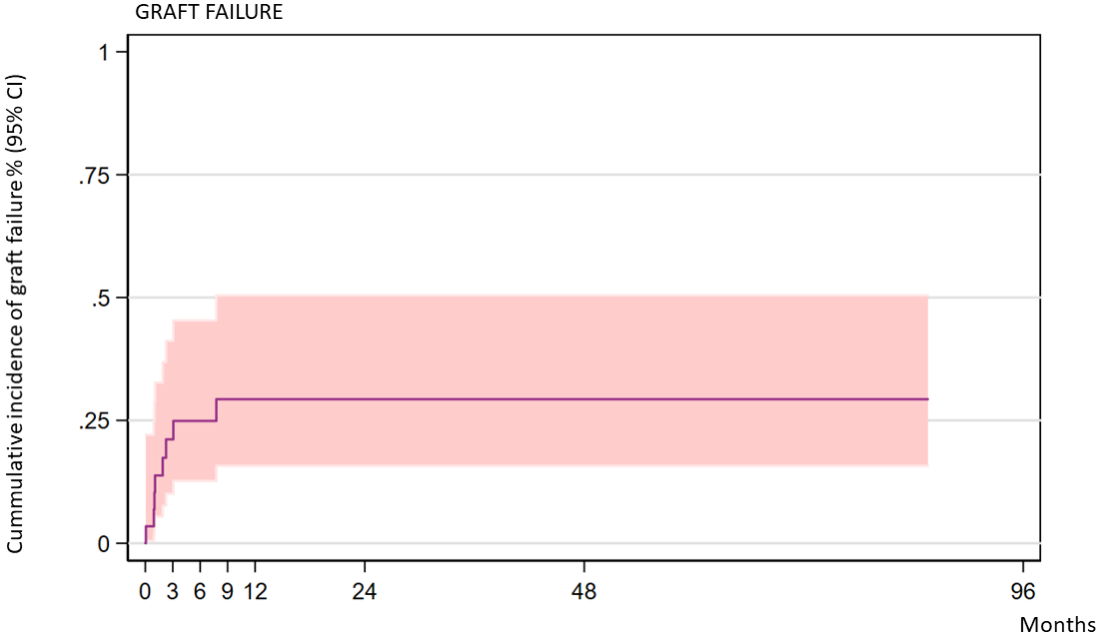
Cumulative incidence of graft failure over time (all patients).

Eight of the 30 transplanted patients experienced graft failure. Two of them had primary graft failure and six had secondary graft rejection. Six of the patients who had graft rejection had received busulfan-based conditioning, and two had received treosulfan-based conditioning. Busulfan AUC was available for one patient (reduced intensity) and was not measured in the other patients. The two patients who experienced primary rejection were 9 years old; the ages of the patients who experienced secondary rejection were 1, 1.3, 2.8, 5.8, 8.9, and 12.3 years. Seven of the patients with graft failure had received transplants from unrelated donors: four patients from 10/10 HLA-matched donors, three from 9/10 HLA-matched donors, and one from an MSD. The cumulative incidence of graft failure at 2 years was 28.26% (CI: 15.15–48.88) (**Figure 1**). The 2-year incidence of graft failure was 11.1% (95% CI 1.64–56.7) for MSD transplants and 37.08% (95% CI 19.33–63.17) for UD transplants (*P* = 0.208). Regarding graft characteristics of the two patients who experienced a primary rejection, one patient had received a graft with less than 2 × 10E8/kg TNC and <2 × 10E6/kg CD34 cells; the second patient had received a graft with partial CD3 depletion.

All patients who experienced primary or secondary rejection underwent a second transplant. After this second procedure, six patients engrafted and two required a third transplant. The stem cell donor for the second transplant was the same as for the first transplant in four patients, a haploidentical donor in three patients, and a different matched unrelated donor in one patient. Of these eight patients, five are engrafted and alive and three died of transplant-related complications.

#### 3.1.2 GVHD

**Figure 2:**
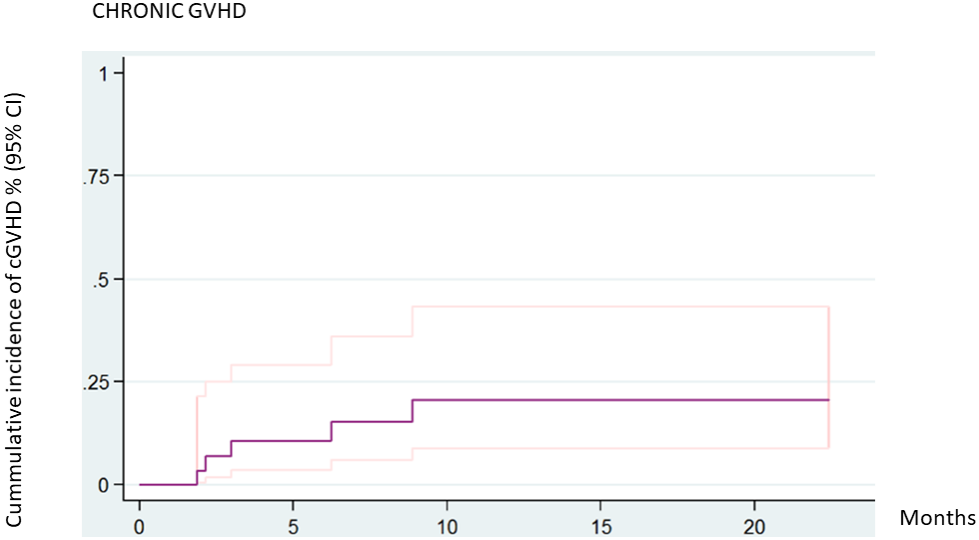
Cumulative incidence of chronic GVHD (all patients).

Acute GVHD ≥grade II was diagnosed in 11 patients and grades III–IV was described in three patients. Chronic GVHD was diagnosed in four patients: the disease was mild in two patients, moderate in one, and severe in one. The 2-year cumulative incidence of cGVHD was 20.5% (95% CI 8.9–43.2). The incidence of cGVHD was 11.1% (95% CI 1.95–56.7) in related donors and 26.71% (95% CI 10.42, 58.44) in unrelated donors (*P* = 0.4811). There were no statistically significant differences in the incidence of cGVHD according to donor type (**Figure 2**).

#### 3.1.3 Other complications

Bacteriemia was diagnosed in nine patients in the first 3 months after HCT.

Viral infections included one CMV disease (colitis), one EBV-PTLD, and one herpes zoster. Four patients had CMV viremia and four patients had more than one virus blood reactivation (without disease): two patients had CMV and EBV, and two patients had HHV6, EBV, CMV, and adenovirus.

Pulmonary aspergillosis was diagnosed in two patients during the period of pancytopenia following graft rejection. No other fungal infections were diagnosed in this cohort.

Two patients experienced post-HCT autoimmune cytopenias. One of them had autoimmune hemolytic anemia that responded to steroids and rituximab. The second patient had autoimmune hemolytic anemia and thrombocytopenia that were steroid-dependent and required abatacept with good response.

### 3.2 Overall survival, graft-failure-free survival, and cGVHD-free survival

**Figure 3:**
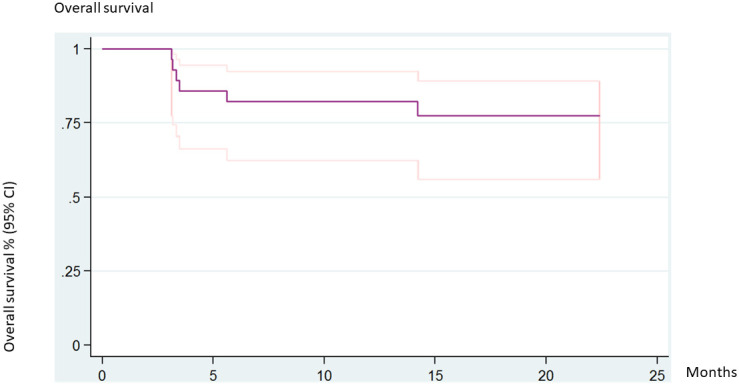
Overall survival (all patients).

The 5-year overall survival rate was 77.3% (95% CI 55.92–89.23). No statistical differences were found when assessing for donor, acute GVHD, or chronic GVHD. Six patients (20%) died of transplant-related causes. The causes of death were acute GVHD, pneumonitis, pulmonary hypertension, venooclusive disease of the liver (VOD), necrotizing hepatitis, and disseminated aspergillosis (**Figure 3**).

**Figure 4:**
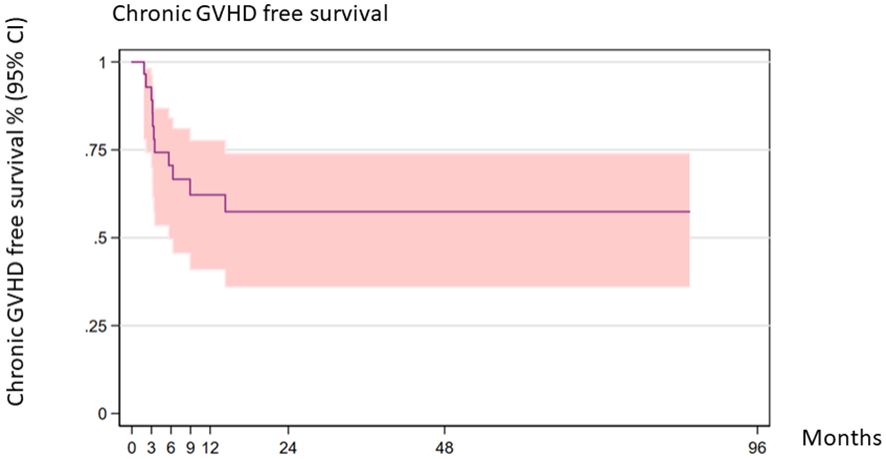
Chronic GVHD-free survival (all patients).

The 2-year cGVHD-free survival was 57.41% (95% CI 36.01–73.97) (**Figure 4**).

The outcomes are summarized in **Table 2**.

**Table 2:** Summary of outcomes.

	Cumulative incidence	95% CI
Graft failure (2 years)	28.26%	15.15–48.8
Graft failure for MSD (2 years)	11.10%	1.64–56.7
Graft failure for UD (2 years)	37.08%	19.33–63.17
Acute GVHD (grades II–IV)	34.19%	14.24–68
Chronic GVHD (2 years)	20.50%	8.9–43.2
Chronic GVHD for MSD (2 years)	11.10%	1.95–56.7
Chronic GVHD for UD (2 years)	26.71%	10.42–58.44
Overall survival (5 years)	77.30%	55.92–89.23
GVHD-free survival (2 years)	57.41%	36.01–73.97
GVHD-free survival for MSD (2 years)	62.50%	22.93–86.07
GVHD-free survival for UD (2 years)	56.05%	30.44–75.44

MSD, matched sibling donor; UD, unrelated donor; GVHD, graft-versus-host disease.

## 4 Discussion

In this report, we describe the transplantation details and outcomes of 30 pediatric patients with chronic granulomatous disease who underwent allogeneic HCT in Spanish pediatric transplant centers. Our results provide useful information for the clinical management and transplantation process of the emerging indication for HCT. However, the relatively small size of the cohort did not allow us to establish statistical significance for factors potentially contributing to adverse events. The main limitations of our report are its retrospective nature and the lack of comparison with the outcome of patients who did not receive HCT.

The small cohort size of our study and the inclusion of siblings may explain the slightly higher proportion of AR inheritance.

The two most common complications were graft failure and GVHD. All patients in the cohort underwent transplantation because of their severe phenotype, characterized by life-threatening infections or inflammatory complications, which may partly account for the high risk of adverse events of graft rejection and GVHD.

HCT is currently the only curative therapy for CGD, but debate continues about which patients are able to benefit and the optimal timing of HCT. In the early 2000s, a few reports of HCT in CGD demonstrated good engraftment in both the MSD and UD settings (4, 11, 12). The patients included in those small series usually received mainly BuCy conditioning, and transplant-related complications were limited to those with pre-existing infection or inflammation. Attempts to reduce the intensity of the conditioning regimen have been published, and the encouraging results have led to a less restrictive approach to transplantation. In 2014, Güngör et al. (13) demonstrated reduced toxicity, a low graft failure rate (5%), and reduced transplant-related mortality in a cohort of 56 patients, mainly with high-risk CGD. These results were confirmed in a large study of 712 patients (635 children) that reported a 3-year OS and EFS of 85.7% and 75.8%, respectively, with a low incidence of cGVHD and a graft failure rate of 12% (14). Overall, these results suggested that a reduced toxicity approach should be preferred in patients with CGD. When treosulfan was used in a cohort of 70 patients (15), the 2-year probability of survival was 90.48% with no significant difference in OS between transplants from UD or MSD. However, secondary graft failure occurred in 12% of the patients.

Previous studies have reported negative outcomes and significant complications associated with the HCT process. For example, a non-myeloablative conditioning regimen consisting of cyclophosphamide and fludarabine resulted in a high rate of graft failure and a transplant-related mortality (TRM) rate as high as 38% (16). In another study, Dedieu et al. (5) compared a cohort of patients who received conventional treatment with a subset of patients who underwent HCT. In this cohort, the graft failure rate was 14% and patients who rejected were either younger than 4 years or had received RIC transplants with busulfan-based conditioning from a 9/10 HLA-matched donor. The results also showed that patients who remained on conventional therapy continued to develop significant complications over time. On the other hand, after successful HCT, there was a reduction in infectious episodes and in the rate of growth retardation. Other countries, such as Japan, have also published their valuable experiences in this setting (17).

Haploidentical HCT in patients with CGD has been shown to be effective in some case reports (18). However, a high rate of GVHD has been reported with the post-HCT cyclophosphamide regimen (19, 20), and the best platform in this setting remains to be determined.

In our cohort, the vast majority of patients with rejection (7/8) had received a transplant from an unrelated donor. Three of the six patients with secondary rejection were younger than 4 years, supporting the idea that this particular subgroup of very young patients may be at higher risk of rejection. Busulfan AUC was available for a minority of patients, which is also a limitation.

Regarding GVHD prophylaxis in our cohort, cyclosporine A plus MMF was the most commonly used regimen. Acute grade III–IV GVHD was described in three patients. GVHD was the cause of death in one patient. In this regard, some reports, at least in the myeloablative setting, describe inferior graft outcomes with cyclosporine plus MMF compared with cyclosporine plus methotrexate (21). Post-HCT infections were usually manageable, but two episodes of pulmonary aspergillosis were diagnosed in the context of graft rejection. Second HCT procedures were feasible in these patients, probably reflecting the fact that these patients were centralized and transplanted in pediatric HCT reference centers. Altogether, these results suggest that the best approach for this group of patients remains to be determined.

In conclusion, allogeneic HCT resulted in the successful resolution of CGD with excellent survival in the majority of patients. These results support the message of feasibility and efficacy of allogeneic HCT in patients with CGD and previous complications, especially in those with a matched sibling donor. However, there are potential challenges in the management of these patients, namely, graft rejection, especially in young patients and those receiving unrelated donor grafts. If a suitable MSD is not available, the decision to proceed to perform HCT with an unrelated donor must be carefully considered and both the platform of the transplant and the conditioning regimen must be refined to try to reduce the risk of rejection and the risk of GVHD. Our results suggest that younger children and children receiving grafts from unrelated donors may require more intensive conditioning to achieve stable engraftment.

## Data availability statement

The original contributions presented in the study are included in the article/**Supplementary Material**. Further inquiries can be directed to the corresponding author.

## Ethics statement

The studies involving humans were approved by Comité Ética Investigación Medicamentos Vall d´Hebron. The studies were conducted in accordance with the local legislation and institutional requirements. Written informed consent for participation was not required from the participants or the participants’ legal guardians/next of kin in accordance with the national legislation and institutional requirements.

## Author contributions

LA: Conceptualization, Writing – original draft, Investigation, Methodology, Supervision. DB: Writing – review & editing, Supervision. JF: Writing – review & editing, Supervision. AR: Writing – review & editing, Supervision. MBB: Writing – review & editing, Supervision. MBC: Writing – review & editing. YM: Writing – review & editing, Supervision. JFS: Writing – review & editing. MU: Writing – review & editing. LS: Writing – review & editing. AP: Writing – review & editing, Supervision, Validation. CD: Conceptualization, Methodology, Supervision, Writing – review & editing.
