# Supplementary material for: Hematopoietic stem cell transplantation in children with chronic granulomatous disease: the Spanish experience
Source: Front Immunol. 2024 Feb 1;15:1307932. doi: 10.3389/fimmu.2024.1307932 (PMC10870648; doi:10.3389/fimmu.2024.1307932)
Supplement: Supplementary file 1 [file Table_1.docx]

Supplementary Table 1. AR: autosomal recessive; PBSC peripehral blood stem cells; n/a not available.. UD: unrelated donor; MSD: matched sibling donor; CsA: cyclosporine A; MTX: methotrexate; MMF: mofetil mycophenolate; HHV6: human herpes virus 6; CMV cytomegalovirus; EBV: epstein barr virus; PTLD: post-HCT lymphoproliferative disease; IFI: invasive fungal infection; VOD: venooclusive disease of the liver; ATG: anti Thymocyte globuline
